# An evaluation of the anti-inflammatory, antipyretic and analgesic effects of hydroethanol leaf extract of *Albizia zygia* in animal models

**DOI:** 10.1080/13880209.2016.1262434

**Published:** 2016-12-07

**Authors:** Wonder Kofi Mensah Abotsi, Stanley Benjamin Lamptey, Stephen Afrane, Eric Boakye-Gyasi, Ruth Uwa Umoh, Eric Woode

**Affiliations:** Department of Pharmacology, Faculty of Pharmacy and Pharmaceutical Sciences, College of Health Sciences, Kwame Nkrumah University of Science & Technology, Kumasi, Ghana

**Keywords:** Pyrexia, pain, inflammation, formalin test, carrageenan, baker’s yeast

## Abstract

**Context:** The leaves of *Albizia zygia* (DC.) J.F. Macbr. (Leguminosae-Mimosoideae) are used in Ghanaian traditional medicine for the treatment of pain, inflammatory disorders and fever (including malaria).

**Objectives:** The present study evaluated the anti-inflammatory, antipyretic and analgesic effects of the hydroethanol leaf extract of *Albizia zygia* (AZE) in animal models.

**Materials and methods:** The anti-inflammatory and antipyretic effects of AZE were examined in the carrageenan-induced foot oedema model and the baker’s yeast-induced pyrexia test respectively. The analgesic effect and possible mechanisms of action were also assessed in the formalin test.

**Results:** AZE (30–300 mg/kg, *p.o*.), either preemptively or curatively, significantly inhibited carrageenan-induced foot edema in 7-day-old chicks (ED_50_ values; preemptive: 232.9 ± 53.33 mg/kg; curative: 539.2 ± 138.28 mg/kg). Similarly, the NSAID diclofenac (10–100 mg/kg, i.p.) significantly reduced the oedema in both preemptive (ED_50_: 21.16 ± 4.07 mg/kg) and curative (ED_50_: 44.28 ± 5.75 mg/kg) treatments. The extract (30–300 mg/kg, p.o.) as well as paracetamol (150 mg/kg, p.o.) also showed significant antipyretic activity in the baker’s yeast-induced pyrexia test (ED_50_ of AZE: 282.5 ± 96.55 mg/kg). AZE and morphine (1–10 mg/kg, i.p.; positive control), exhibited significant analgesic activity in the formalin test. The analgesic effect was partly or wholly reversed by the systemic administration of naloxone, theophylline and atropine.

**Conclusion:** The results suggest that AZE possesses anti-inflammatory, antipyretic and analgesic properties, which justifies its traditional use. Also, the results show the involvement of the opioidergic, adenosinergic and the muscarinic cholinergic pathways in the analgesic effects of AZE.

## Introduction

*Albizia zygia* (DC.) J.F. Macbr. (Leguminosae-Mimosoideae) is a gum-producing, medium-sized deciduous tree widely found in tropical Africa. It is commonly known as *okuro* in Ghana. In African traditional medicine, the leaves are used to treat fever, malaria, diarrhoea, oedema, conjunctivitis and sexual impotence (Kerharo & Bouquet 1950; Adjanohoun et al. 1989; Jiofack et al. 2009). In previous phytochemical evaluation, lupen-20(30)-3β-ol, 14α-stigmast-5-en-3β-ol and 5α-stigmast-7,22-dien-3β-ol were isolated from the bark of *Albizia zygia* (Schoppa & Pachaly 1981). The same compounds were isolated from the leaves and, in addition, a glycoside of lupen-20(30)-3β-ol as well as four other compounds (Schoppa & Pachaly 1981). Albiziaprenol and phytol as well as three flavonoids [4′,7-dihydroxyflavanone; 3′,4′,7-trihydroxyflavone; 3-*O*-methylfisetin (3′,4′,7-trihydroxy-3-methoxyflavone)] have also been isolated from the bark (Pachaly et al. 1983; Abdalla & Laatsch 2012). The gum from the bark of the plant has been widely investigated for its chemical and physical properties in comparison with other mucilages (Ashton et al. 1975; Mital et al. 1978; Odeku 2005).

Few pharmacological studies have been carried out on *A. zygia*. The methanol stem bark extract was found to have antioxidant and analgesic properties (Oloyede & Ogunlade 2013; Abere et al. 2014). The same extract was also active against *Plasmodium falciparum* K1 strain and *Trypanosoma brucei rhodesiense* (Ndjakou Lenta et al. 2007) as well as some bacteria strains (Oloyede & Ogunlade 2013). Literature search has revealed that there are no scientific reports to support the effectiveness of the leaves of *Albizia zygia* in the treatment of pain, inflammation and fever. This study, therefore, evaluated the anti-inflammatory, antipyretic and analgesic effects of the hydroethanol leaf extract of the plant in animal models.

## Materials and methods

### Plant collection and extraction

The leaves of *Albizia zygia* were collected from the campus of Kwame Nkrumah University of Science and Technology (KNUST), Kumasi, Ghana (6°40′31.8″N 1°34′44.1″W), in September 2013 and authenticated by Dr. George Henry Sam of the Department of Herbal Medicine, Faculty of Pharmacy and Pharmaceutical Sciences, Kwame Nkrumah University of Science and Technology (KNUST), Kumasi, Ghana. A voucher specimen (KNUST/H/M/2016/R001) was kept at the herbarium of the faculty.

The leaves were room-dried and pulverized into fine powder with a hammer mill. The powdered leaves (1 kg) were extracted by cold maceration with 70% (^v^/_v_) ethanol and then concentrated under reduced pressure at 50 °C into a syrupy mass in a rotary evaporator. The extract was further dried in a hot air oven at 50 °C for a week and then kept in a refrigerator for later use. The final yield was 16.8% (^w^/_w_). This crude extract is subsequently referred to as *Albizia zygia* extract (AZE) or extract in this study.

### Drugs and chemicals

The following drugs and chemicals were used: formalin and theophylline (BDH, Poole, England); diclofenac (KRKA^®^, Novo Mesto, Slovenia); morphine, paracetamol (PhytoRiker^®^, Accra, Ghana); λ-carrageenan, atropine, naloxone (Sigma-Aldich Inc., St. Louis, MO); commercially available dried baker’s yeast (*Saccharomyces cerevisiae*, Saf do Brasil Produtos Limenticios Ltd, Brazil). The extract was suspended in 2% tragacanth and administered orally.

### Animals

Male Sprague–Dawley rats (100–200 g) were used in the experiments. The animals were purchased from Noguchi Memorial Institute for Medical Research, Accra, Ghana and kept at the animal house of the Department of Pharmacology, KNUST, Kumasi. Animals were housed in stainless steel cages (34 cm ×47 cm ×18 cm; 5 rats per cage) with soft wood shavings as bedding. The animals were maintained under laboratory conditions (temperature 24–28 °C, relative humidity 60–70%, 12 h light–dark cycle) with free access to solid pellet diet (Agricare Ltd, Kumasi, Ghana) and water.

Cockerels (*Gallus gallus;* strain Shaver 579, Akropong Farms, Kumasi, Ghana) were obtained one-day post-hatch and housed in stainless steel cages (34 cm ×57 cm ×40 cm) at a population density of 12–13 chicks per cage. Food (Agricare Ltd, Kumasi, Ghana) and water were available *ad libitum*. Overhead incandescent illumination was provided with room temperature at 29 °C. Chicks were tested at 7 days of age. All experiments were conducted in accordance with the guidelines concerning the care and use of laboratory animals in experimentation (Directive 2010/63/EU). All protocols used were approved by the Departmental Ethics Committee.

### Acute toxicity test

Rats were divided into four groups (*n* = 5) and fasted overnight, but provided with water *ad libitum*. On the test day, rats were orally treated with AZE (300, 1000 and 3000 mg/kg) or vehicle (10 mL/kg). Animals were then monitored for gross behavioural changes and mortality at 0, 15, 30, 60, 120 and 180 min, and also at 24 h post-extract administration. The rats were also observed daily for up to 14 days to detect any possible delayed deaths.

### Carrageenan-induced oedema in chicks

The anti-inflammatory activity of AZE was assessed using the carrageenan-induced foot oedema in the chick (Roach & Sufka 2003; Abotsi et al. 2012). Oedema was induced by injecting carrageenan (10 μL of a 2% solution in saline) into the sub-plantar tissue of the right footpads of the chicks. Foot volume was measured before carrageenan injection and at hourly intervals over 5 h by water displacement (Fereidoni et al. 2000). The foot oedema was quantified by measuring the percentage change in foot volume over the various time points.

Two sets of experiments were carried out to assess the anti-inflammatory activity of AZE. The first was to study the effect of the drugs administered preemptively (30 min for i.p. route and 1 h for oral route) before the carrageenan injection. The second examined the effects of the drugs given 1 h post carrageenan injection. Groups of chicks (*n* = 6) were treated with AZE suspended in 2% tragacanth (30–300 mg/kg, p.o.). Diclofenac (10–100 mg/kg, i.p.) was used as a standard and the drug vehicle (2% tragacanth, 10 mL/kg, p.o.) served as a control. Drug effects were assessed by comparing either the peak oedema response attained during the 5 h or the total oedema (monitored as area under the time course curves) response developed during the period.

### Antipyretic test

Rectal temperature (*T*_R_) was recorded by inserting a lubricated digital thermometer (external diameter: 3 mm, 0.1 °C precision) 3 cm into the rectum of rats. Animals presenting initial rectal temperature (*T*_R_) between 36 and 37 °C were selected for the antipyretic tests. The effect of drugs on baker’s yeast-induced hyperthermia were then determined as previously described (Tomazetti et al. 2005; Boakye-Gyasi et al. 2011). After measuring basal *T*_R_ of the animals, they were injected with a pyrogenic dose of baker’s yeast (0.135 g/kg, i.p.). *T*_R_ changes were recorded every hour up to 4 h. Animals that showed an increase of not less than 0.5 °C in rectal temperature were selected for the experiment. Animals were randomly divided into five groups of five rats each. Group 1 received 2% tragacanth suspension (10 mL/kg, p.o.). The rest of the groups received AZE (30, 100, 300 mg/kg; p.o.) or the antipyretic, paracetamol (150 mg/kg, p.o.). There was a sixth group that received only normal saline (0.9% NaCl, i.p.); baker’s yeast was not administered. *T*_R_ were monitored hourly over the following 4 h period after extract/drugs administration.

### Formalin-induced nociception

The anti-nociceptive effect of the extract was evaluated using the formalin test. The test was carried out as described previously (Dubuisson & Dennis 1977; Woode et al. 2011). Rats were randomly divided into seven groups (*n* = 5) and each animal acclimatized to the testing environment (Perspex chamber, 30 cm ×30 cm ×30 cm) for 1 h. Rats were pretreated with AZE (30, 100 and 300 mg/kg, p.o.), morphine (1, 3 and 10 mg/kg, i.p.) or vehicle (10 mL/kg, p.o.) 30 min (i.p.) or 1 h (p.o.) before intraplantar injection of 50 μL of 2.5% formalin. Animals were immediately returned individually into the testing chamber. A mirror angled at 45° beneath the floor of the chamber allowed an unobstructed view of all the paws. The behaviour of the animals was then captured (1 h) for analysis by a camcorder (Everio™ model GZ-MG1300, JVC, Tokyo, Japan) attached to a computer and placed directly opposite the mirror. Pain responses were scored for 1 h, starting immediately after formalin injection. A nociceptive score was determined for each 5-min time block by measuring the amount of time spent and frequency of biting/licking of the injected paw (Hayashida et al. 2003). The scoring of pain responses was done with the aid of the public domain software JWatcher™ Version 1.0 (University of California, Los Angeles, USA and Macquarie University, Sydney, Australia available at http://www.jwatcher.ucla.edu/). Average nociceptive score for each time block was calculated by multiplying the frequency and time spent in biting/licking. Data were expressed as the mean ± S.E.M. scores between 0–10 and 10–60 min after formalin injection.

### Possible mechanism of action of AZE in the formalin test

The effects of AZE (100 mg/kg, p.o.), morphine (3 mg/kg, i.p) or vehicle (10 mL/kg, p.o.) were evaluated in the presence of naloxone (a nonselective opioid receptor antagonist; 2 mg/kg, i.p), theophylline (a nonselective adenosine receptor antagonist; 10 mg/kg, i.p,) and atropine (a nonselective muscarinic receptor antagonist; 5 mg/kg, i.p) to determine the involvement of the opioidergic, adenosinergic and muscarinic cholinergic systems respectively in their anti-nociceptive activity. The antagonists were given 15 min before the administration of AZE, morphine or vehicle. The nociceptive response to formalin injection was recorded 1 h after the administration of AZE or vehicle and 30 min after the administration of morphine. The doses of antagonists and other drugs were selected on the basis of previous literature data and in pilot experiments in the laboratory (Woode & Abotsi 2011; Woode et al. 2012).

### Data analysis

All data are presented as mean ± S.E.M. The time-course curves were subjected to two-way (*treatment × time*) repeated measures analysis of variance (ANOVA) with Tukey’s *post hoc* test. Total oedema, total change in *R*_T_ or total nociceptive score for each treatment was calculated in arbitrary unit as the area under the curve (AUC). Differences in AUCs were analyzed using one-way ANOVA, with drug treatment as a between-subjects factor, followed by Tukey’s multiple comparison test. GraphPad Prism for Windows, Version 5 (GraphPad Software, San Diego, CA) was used for all statistical analyses and ED_50_ determinations. *p* < 0.05 was considered statistically significant in all analyses.

## Results

### Acute toxicity test

No toxic signs or mortality were observed during the study period of 14 days.

### Carrageenan-induced oedema

**Figure 1.**
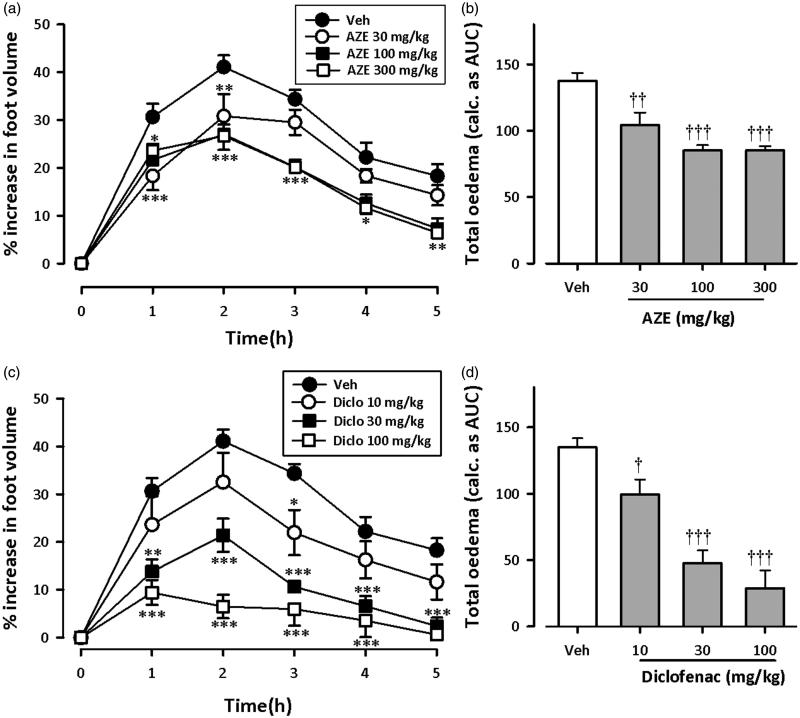
Effect of preemptive treatment of chicks with AZE (30–300 mg/kg; p.o.) and diclofenac (10–100 mg/kg, i.p.) on time course curves (a, c) and the total oedema response (b, d) in carrageenan-induced foot oedema. Values are means ± S.E.M. (*n* = 6). *p < 0.05; **p < 0.01; ***p < 0.001 compared to vehicle-treated group (Two-way ANOVA followed by Tukey’s multiple comparison test). †*p* < 0.05; ††*p* < 0.01; †††*p* < 0.001 compared to vehicle-treated group (One-way ANOVA followed by Tukey’s multiple comparison test).

**Figure 2.**
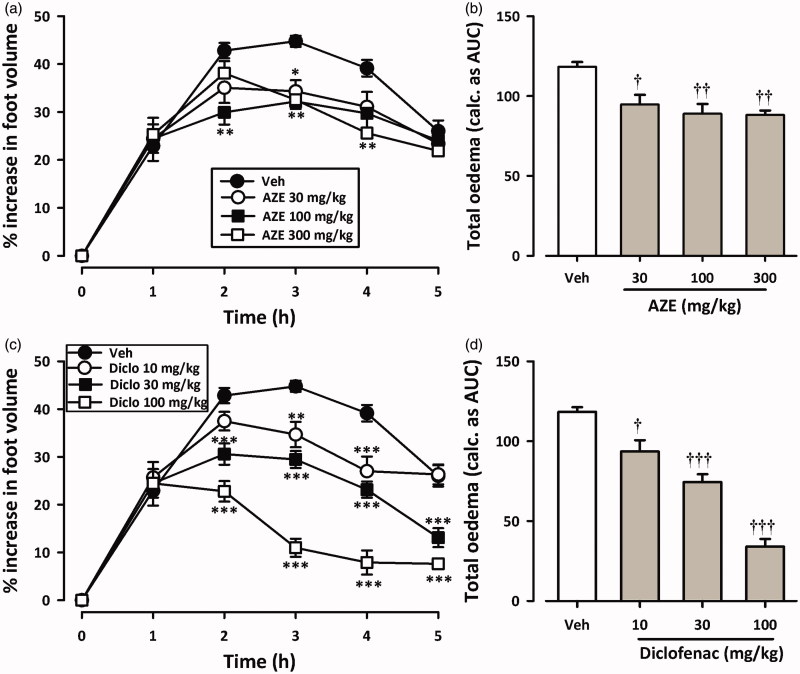
Effect of AZE (30–300 mg/kg, p.o.) and diclofenac (10–100 mg/kg, i.p.) on time-course curves (a, c) and the total oedema response (b, d) in the curative protocol of carrageenan-induced foot oedema in chicks. Values are means ± S.E.M. (*n* = 6). *p < 0.05; **p < 0.01; ***p < 0.001 compared to vehicle-treated group (Two-way ANOVA followed by Tukey’s multiple comparison test). †*p* < 0.05; ††*p* < 0.01; †††*p* < 0.001 compared to vehicle-treated group (One-way ANOVA followed by Tukey’s multiple comparison test).

Figures 1(a,c) and 2(a,c) show the time course curves for effects of AZE and diclofenac on carrageenan-induced oedema in chicks. Carrageenan injection (10 μL, 2% suspension) induced moderate inflammation resulting in foot oedema in the 7-day-old chicks peaking at 2–3 h (Figures 1 and 2), as described by Roach and Sufka (2003). Two-way ANOVA (*treatment × time*) revealed a significant effect of drug treatment for AZE (preemptive: *F*_3_,_18 _=_ _15.95, *p* < 0.0001; curative: *F*_3_,_18 _=_ _5.44, *p* = 0.0077) and diclofenac (preemptive: *F*_3_,_20 _=_ _32.54, *p* < 0.0001; curative: *F*_3_,_19 _=_ _31.42, *p* < 0.0001). Total oedema produced by each treatment is expressed in arbitrary units as AUC of the time-course curves. AZE (30–300 mg/kg, p.o.) significantly reduced foot oedema with maximal inhibition of 38.12 ± 2.92% and 25.54 ± 2.36% for preemptive (Figure 1(b)) and curative (Figure 2(b)) treatments, respectively. Similarly, the NSAID diclofenac (10-100 mg/kg, i.p.) reduced the oedema by a maximum of 81.23 ± 8.30% and 71.23 ± 3.98%, respectively, for preemptive (Figure 1(d)) and curative treatments (Figure 2(d)).

From the ED_50_ values obtained on non-linear regression analysis of dose–response curves, AZE (preemptive: 232.9 ± 53.33 mg/kg; curative: 539.2 ± 138.28 mg/kg) was less potent than diclofenac (preemptive: 21.16 ± 4.07 mg/kg; curative: 44.28 ± 5.75 mg/kg). Also, AZE was more effective at inhibiting foot oedema when given preemptively than curatively.

### Antipyretic test

**Figure 3.**
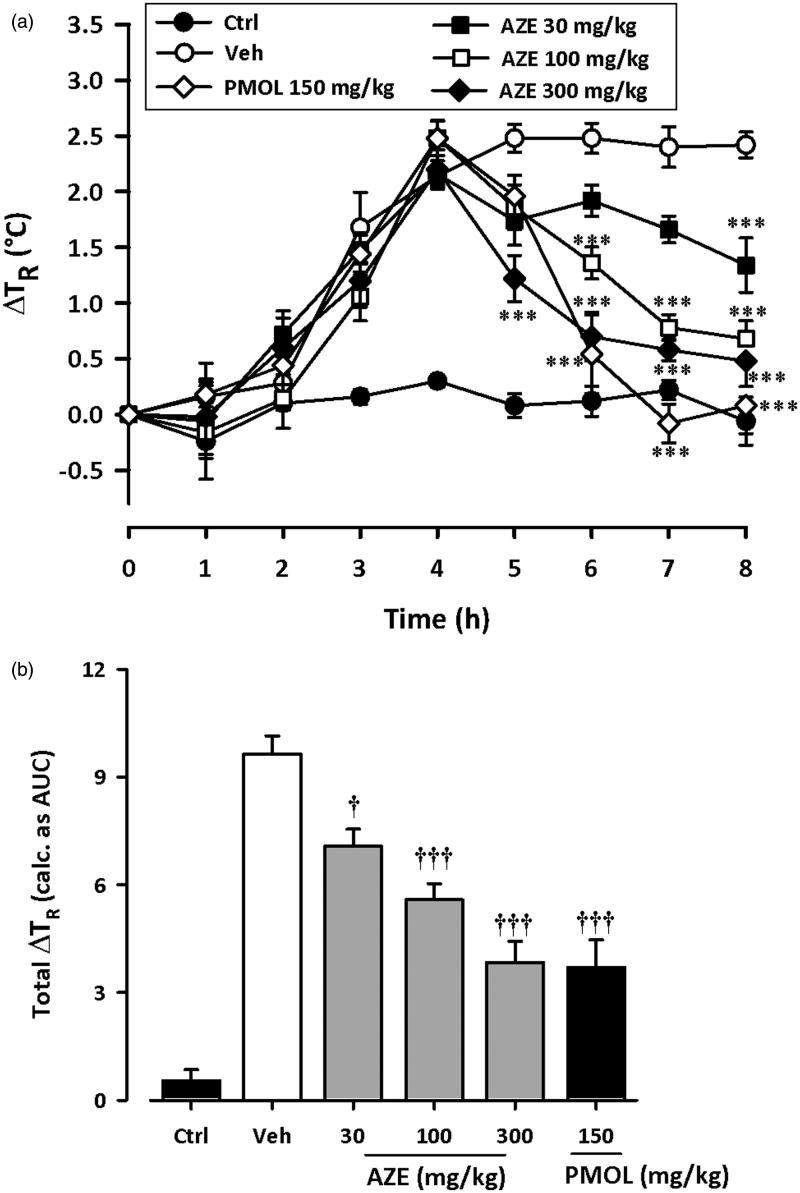
Effect of AZE (30–300 mg/kg, p.o.) and paracetamol, PMOL (150 mg/kg, p.o.) on time-course curve (a) and the total change in temperature (calculated as AUCs) (b) on baker’s yeast-induced changes of rectal temperatures in rats. Control (Ctrl) represents naive animals (no treatment with yeast). Values are means ± S.E.M. (*n* = 5). ***p < 0.001 compared to vehicle-treated group (Two-way ANOVA followed by Tukey’s multiple comparison test). †*p* < 0.05; †††*p* < 0.001 compared to vehicle-treated group (One-way ANOVA followed by Tukey’s multiple comparison test).

Figure 3(a) shows the time course curves for effects of AZE and paracetamol on baker’s yeast-induced pyrexia in rats. Intraperitoneal injection of yeast caused a steady incremental change in rectal temperature of rats, peaking at about 4 h (Figure 3(a)).

Considering the AUCs, ANOVA revealed significant effect of AZE (30–300 mg/kg) on yeast-induced pyrexia (*F*_5_,_24 _=_ _34.85, *p* < 0.0001). Follow-up analysis with Tukey’s multiple comparison test revealed statistical significance at doses 30 mg/kg (*p* < 0.05), 100 mg/kg (*p* < 0.001) and 300 mg/kg (*p* < 0.001) (Figure 3(b)). AZE (30–300 mg/kg, p.o.) significantly reduced pyrexia with maximal inhibition of 60.17 ± 6.03% at the dose of 300 mg/kg. The administration of the antipyretic paracetamol (150 mg/kg, p.o.) at 4 h, also significantly attenuated the change in rectal temperature (*F*_5,24 _=_ _34.85, *p* < 0.0001) as depicted in the time course curves and AUCs (Figure 3). Paracetamol at the dose of 150 mg/kg inhibited pyrexia by 61.62 ± 7.88%. The ED_50_ value obtained for AZE was 282.5 ± 96.55 mg/kg.

### Formalin test

**Figure 4.**
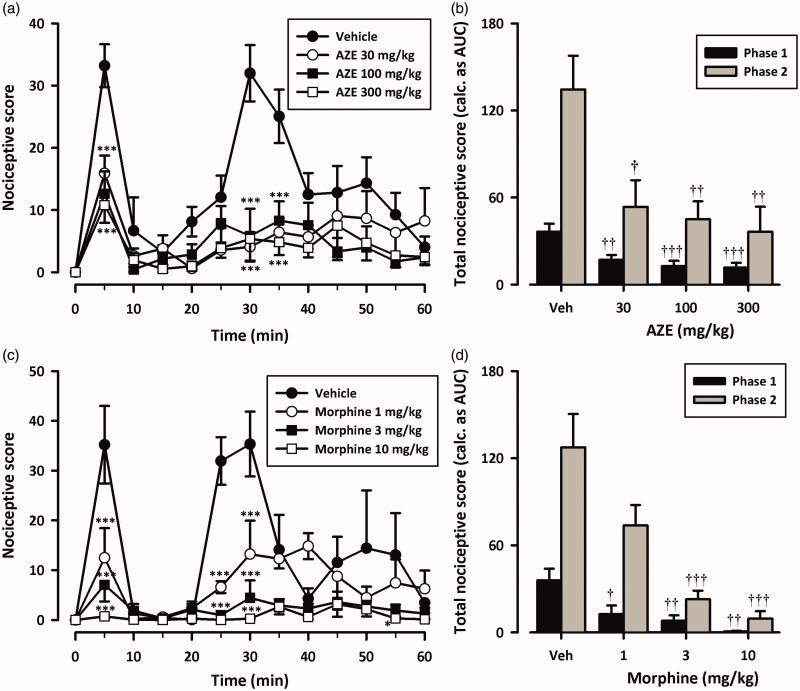
Dose–response effects of AZE (30–300 mg/kg, p.o.) (a, b) and morphine (1–10 mg/kg, i.p.) (c, d) on formalin-induced nocifensive behaviours in rats. Left panels show the time course of effects over the 60 min period and the right panels show the total nociceptive score calculated from AUCs over the first (0-10 min) and second (10–60 min) phases. Nociceptive scores are shown in 5 min time blocks up to 60 min post-formalin injection. Values are means ± S.E.M. (*n* = 5). *p < 0.05; ***p < 0.001 compared to vehicle-treated group (Two-way ANOVA followed by Tukey’s multiple comparison test). †*p* < 0.05; ††*p* < 0.01; †††*p* < 0.001 compared to vehicle-treated group (One-way ANOVA followed by Tukey’s multiple comparison test).

Figure 4 shows the effect of pretreatment of AZE and morphine on formalin-induced pain in rats. Injection of formalin (2.5%, 50 μL) into the ventral surface of the right hind paw evoked a characteristic biphasic licking response in the rats as previously reported (Wheeler-Aceto et al. 1990; Abbott et al. 1995). All drug-treated groups displayed significant reduction in formalin-induced nociceptive behaviour (Figure 4(a,c)) when compared with the vehicle-treated group [(AZE: *F*_3,16 _=_ _7.36; *p =* 0.0006; morphine: *F*_3,16 _=_ _17.82; *p* < 0.0001; Two-way ANOVA (*treatment* ×* time*)]. Oral administration of AZE (30–300 mg/kg) 1 h before the injection of formalin inhibited both neurogenic (*F*_3,16 _=_ _8.45; *P =* 0.0002, Figure 4(b)) and inflammatory (*F*_3,16 _=_ _6.22; *p =* 0.0016, Figure 4(b)) phases of formalin-induced licking with maximal inhibition of 67.81 ± 8.73% and 72.85 ± 12.74%, respectively. Morphine (1–10 mg/kg, i.p.), the positive analgesic control, similarly produced marked dose-related inhibition of both the early (*F*_3,16 _=_ _8.05, *p* = 0.0017, Figure 4(d)) and late (*F*_3,16 _=_ _14.73, *p* < 0.0001, Figure 4(d)) phases. Morphine reduced formalin-evoked nocifensive behaviours by 97.93 ± 1.17% and 92.52 ± 3.96% respectively in the neurogenic and the inflammatory phases of the formalin test (Figure 4(d)). The ED_50_ values for AZE were 44.67 ± 12.95 mg/kg, 33.06 ± 12.58 mg/kg for phase 1 and 2, respectively. Morphine was more potent in both phases (0.60 ± 0.23 mg/kg, 1.04 ± 0.23 mg/kg).

### Assessment of the possible mechanism of action of AZE

**Figure 5.**
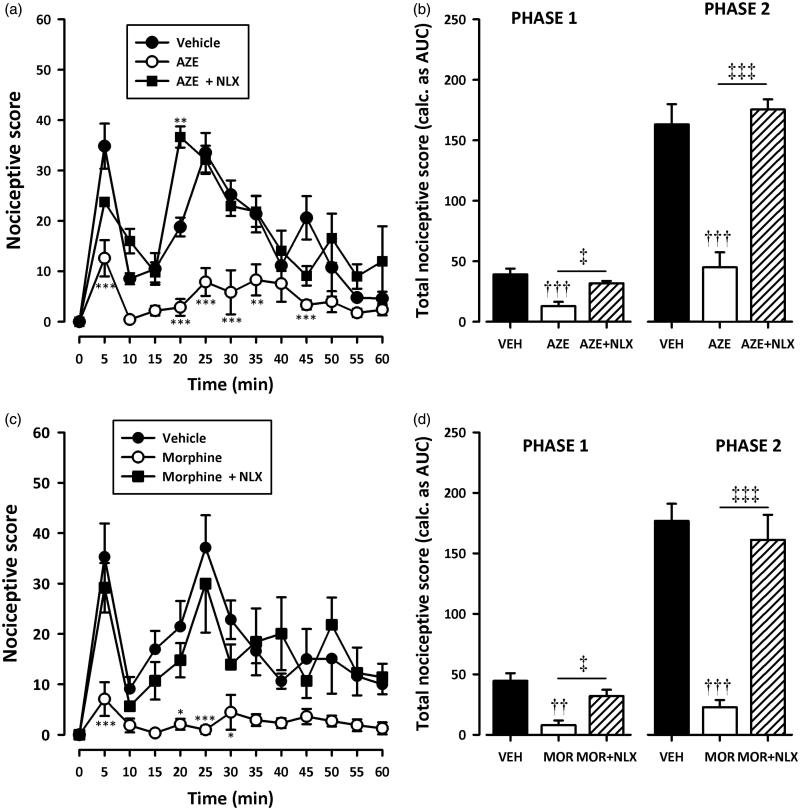
Effect of pretreatment of rats with naloxone on the anti-nociceptive effects of AZE (100 mg/kg, p.o.) (a, b) and morphine (3 mg/kg, i.p.) (c, d) in the formalin test. Left panels show the time course of effects over the 60 min period and the right panels show the total nociceptive score calculated from AUCs over the first (0–10 min) and second (10–60 min) phases. Values are means ± S.E.M. (*n* = 5). *p < 0.05; **p < 0.01; ***p < 0.001 compared to vehicle-treated group (Two-way ANOVA followed by Tukey’s multiple comparison test). ††*p* < 0.01; †††*p* < 0.001 compared to vehicle-treated group; ‡*p* < 0.05; ‡‡‡*p* < 0.001 compared to AZE 100 mg/kg or morphine 3 mg/kg (One-way ANOVA followed by Tukey’s multiple comparison test).

**Figure 6.**
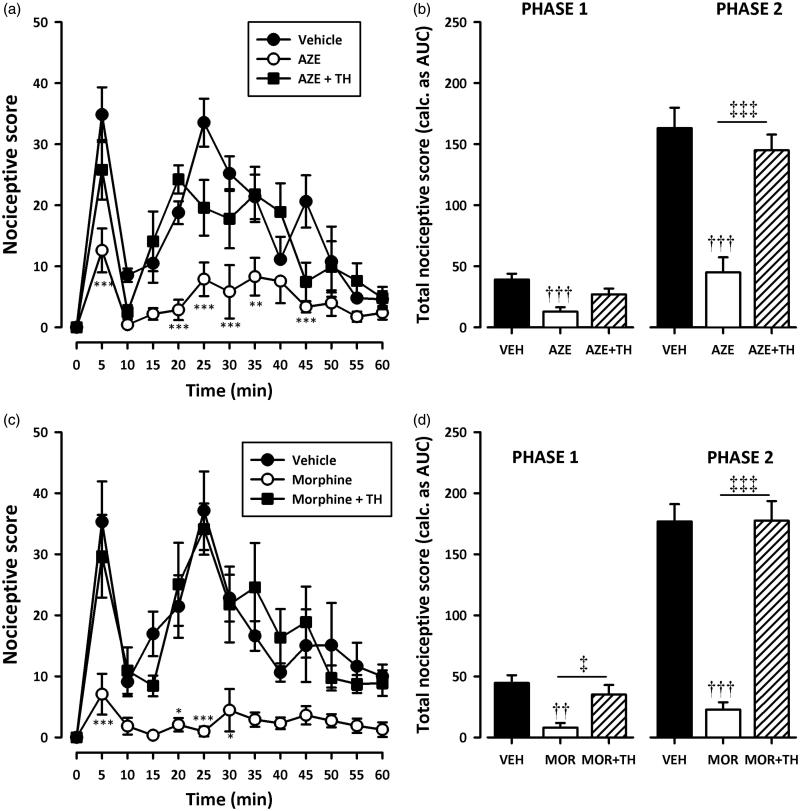
Effect of pretreatment of rats with theophylline on the anti-nociceptive effects of AZE (100 mg/kg, p.o.) (a, b) and morphine (3 mg/kg, i.p.) (c, d) in the formalin test. Left panels show the time course of effects over the 60 min period and the right panels show the total nociceptive score calculated from AUCs over the first (0–10 min) and second (10–60 min) phases. Values are means ± S.E.M. (*n* = 5). *p < 0.05; **p < 0.01; ***p < 0.001 compared to vehicle-treated group (Two-way ANOVA followed by Tukey’s multiple comparison test). ††*p* < 0.01; †††*p* < 0.001 compared to vehicle-treated group; ‡*p* < 0.05; ‡‡‡*p* < 0.001 compared to AZE 100 mg/kg or morphine 3 mg/kg (One-way ANOVA followed by Tukey’s multiple comparison test).

**Figure 7.**
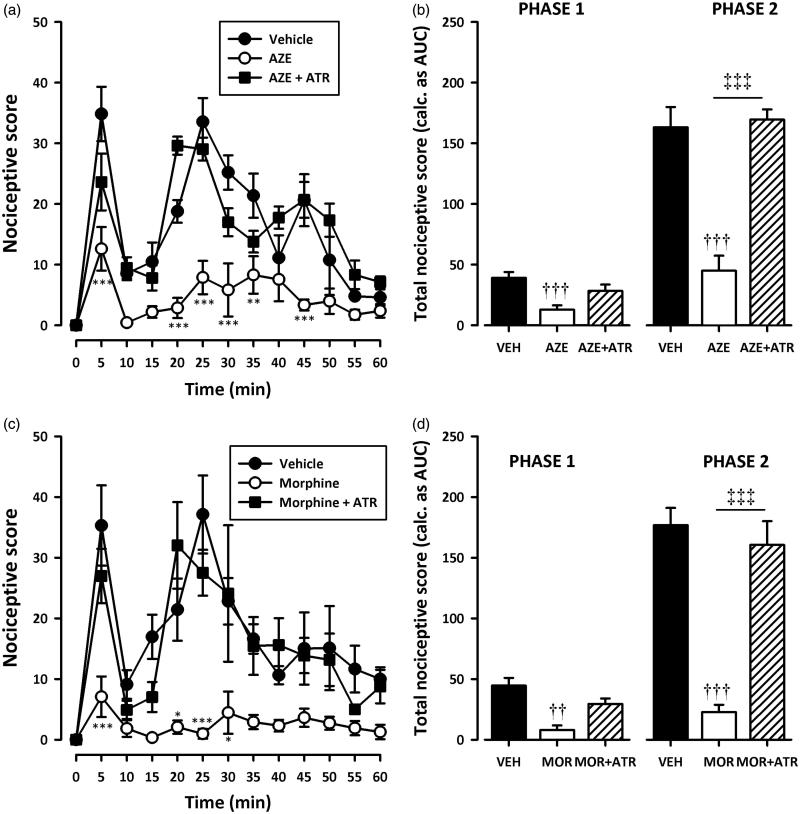
Effect of pretreatment of rats with atropine on the anti-nociceptive effects of AZE (100 mg/kg, p.o.) (a, b) and morphine (3 mg/kg, i.p.) (c, d) in the formalin test. Left panels show the time course of effects over the 60 min period and the right panels show the total nociceptive score calculated from AUCs over the first (0–10 min) and second (10–60 min) phases. Values are means ± S.E.M. (*n* = 5). *p < 0.05; **p < 0.01; ***p < 0.001 compared to vehicle-treated group (Two-way ANOVA followed by Tukey’s multiple comparison test). ††*p* < 0.01; †††*p* < 0.001 compared to vehicle-treated group; ‡‡‡*p* < 0.001 compared to AZE 100 mg/kg or morphine 3 mg/kg (One-way ANOVA followed by Tukey’s multiple comparison test).

Pretreatment of rats with naloxone (2 mg/kg, i.p), theophylline (10 mg/kg, i.p) or atropine (5 mg/kg, i.p) significantly (all *p* < 0.001) inhibited the analgesic effect of AZE and morphine in the second phase of the formalin test (Figures 5–7). Naloxone also significantly (all *p* < 0.05) inhibited the anti-nociception caused by AZE and morphine (Figure 5) in the first phase of the formalin test. However, the attenuation of analgesia caused by pretreatment of AZE and morphine with atropine in the neurogenic phase did not reach statistical significance (Figure 7). Theophylline significantly blocked the anti-nociceptive effect of morphine in the first phase (*p* < 0.05) but its inhibition of the anti-nociceptive effect of AZE did not reach statistical significance (*p* > 0.05).

## Discussion

The leaves of *Albizia zygiza* have been used in African traditional medicine to treat various pain and inflammatory conditions. It has also been widely reported to be useful in the treatment of fever and malaria. The present study establishes the anti-inflammatory, anti-pyretic and analgesic properties of the hydroethanol leaf extract of *Albizia zygia* in animal models. Preliminary acute toxicity assessment of the leaf extract in rats revealed no deaths after 14 days of observation suggesting that the LD_50_ of the extract is above 3000 mg/kg.

The anti-inflammatory effect of AZE was assessed in the carrageenan-induced oedema test (Winter et al. 1962), a classical model of acute inflammation that has been extensively used to screen new anti-inflammatory drugs (Di Rosa & Willoughby 1971). The carrageenan-induced oedema has been validated in chicks (Roach & Sufka 2003), and is much more economical than rodent models. The chick model was therefore used in this study. Oral administration of AZE significantly inhibited oedema induced by carrageenan in both preemptive and curative protocols of the anti-inflammatory activity assessment. The finding justifies the folkloric use of the plant extract in the treatment of inflammatory conditions. According to Vinegar et al. (1969), the inflammatory response induced by carrageenan is biphasic. First (early) phase is characterized by marked oedema formation resulting from the rapid production of several inflammatory mediators including histamine, serotonin and bradykinin (Di Rosa et al. 1971). This is subsequently sustained by the release of prostaglandins and nitric oxide (second phase) with peak at 3 h, produced by inducible isoforms of COX (COX-2) and nitric oxide synthase (iNOS), respectively (Seibert et al. 1994; Salvemini et al. 1996). Clinically effective anti-inflammatory drugs are most effective against the second (late) phase (Vinegar et al. 1969; Di Rosa & Willoughby 1971). Even though the exact anti-inflammatory mechanisms are yet to be established, it is possible that AZE acts through the inhibition of the release and/or action of inflammatory mediators (e.g. histamine, serotonin, bradykinin, prostaglandins and other cyclooxygenase products) since it inhibited both early and late phases of oedema.

Baker’s yeast-induced fever model, as described earlier by Tomazetti et al. (2005), is a low-cost and reliable method for inducing fever in young rats. The method induces a clear-cut fever, which is reverted by antipyretics commonly used in human beings and selected novel antipyretics in small animals (Tomazetti et al. 2005). From the results obtained, *Albizia zygia* hydroethanol leaf extract possesses significant antipyretic effect comparable to paracetamol. It is well documented that the metabolism of arachidonic acid via cyclooxygenases and, in turn, production of prostaglandins, particularly PGE_2_, is an essential pathway for the generation of fever (Kozak et al. 2000; Aronoff & Neilson 2001; Walter et al. 2016). Therefore, the reduction in the baker’s yeast-induced fever by the extract in this study suggests some influence on prostaglandin biosynthesis and/or action. The results support the traditional use of *A. zygia* leaves in the treatment of fever (including fever associated with malaria).

The analgesic properties of AZE were evaluated in the formalin-induced pain model. The model, originally described by Dubuisson and Dennis (1977), is undoubtedly the most predictive of acute pain and very popular for the rapid and easy screening of pharmacological targets in drug evaluation (Hunskaar & Hole 1987; Tjolsen et al. 1992). The formalin test has two different phases, reflecting different types of pain. A first phase (neurogenic pain), occurring within seconds of formalin injection, is elicited by direct chemical activation of nociceptive primary afferent fibres. A second, later phase (inflammatory pain), reflecting ongoing activity in primary afferents and central sensitization of spinal cord circuits secondary to the barrage of input that occurs during the first phase (Dubuisson & Dennis 1977; Tjolsen et al. 1992; Coderre et al. 1993; McNamara et al. 2007; Shields et al. 2010). According to earlier studies (Hunskaar & Hole 1987; Shibata et al. 1989; Zhang et al. 2015), centrally acting drugs, such as opioids, inhibit both phases of the formalin test equally while many NSAIDs and corticosteroids inhibit only the second phase. In the current study, AZE inhibited both phases of the formalin test suggesting that it is effective against both neurogenic and inflammatory pain. The inhibition of the second phase also confirms the anti-inflammatory effect of the extract.

Some possible anti-nociceptive mechanisms of AZE were investigated with antagonists (naloxone, theophylline and atropine) in the formalin test. Naloxone, a nonselective opioid antagonist, significantly reversed the anti-nociceptive effect of AZE in both phases of the formalin-induced nociception suggesting a possible opioidergic involvement in the actions of AZE. Theophylline, a nonselective adenosine receptor antagonist, also inhibited the analgesic effects of AZE implicating the involvement of adenosinergic pathway in its actions. The involvement of adenosine in morphine anti-nociception is well known (Sawynok 1998; Trang et al. 2015) and has been confirmed in this study. The attenuation of the anti-nociceptive effects of AZE by the nonselective muscarinic receptor antagonist, atropine implicates the muscarinic cholinergic system in the actions of the extract.

The current study clearly establishes the anti-inflammatory, antipyretic and analgesic properties of the leaf extract of *A. zygia*. The presence of lupen-20(30)-3β-ol (lupeol), 14α-stigmast-5-en-3β-ol (β-sitosterol) and 5α-stigmast-7,22-dien-3β-ol (α-spinasterol) in the leaves of the plant (Pachaly et al. 1983; Schoppa & Pachaly 1981) may account, at least in part, for the observed pharmacological effects. Several studies have shown that lupeol, a phytosterol and triterpene, has anti-inflammatory and anti-nociceptive properties (Fernandez et al. 2001; Geetha & Varalakshmi 2001; Saleem 2009; Lucetti et al. 2010; Siddique & Saleem 2011; de Lima et al. 2013). The anti-inflammatory, anti-nociceptive and antipyretic effects of β-sitosterol are also well established (Gupta et al. 1980; Nirmal et al. 2012; Liz et al. 2013; Acikara et al. 2014). Additionally, α-spinasterol, an antagonist of the transient receptor potential vanilloid 1 (TRPV1) receptor, has anti-nociceptive and anti-inflammatory properties (Zhou et al. 1985; Jeong et al. 2004; Trevisan et al. 2012; Borges et al. 2014). Further studies are, however, required to establish all the active constituents in the leaves as well as the mechanisms of the observed pharmacological effects.

## Conclusion

In conclusion, the hydroethanol leaf extract of *Albizia zygia* possess anti-inflammatory, antipyretic and analgesic properties. This validates its traditional uses in the management of fever, pain and inflammatory conditions. Also, the results show the involvement of the opioidergic, adenosinergic and the muscarinic cholinergic pathways in the analgesic effects of AZE.
